# A Monte Carlo Study of the Early Steps of Functional Amyloid Formation

**DOI:** 10.1371/journal.pone.0146096

**Published:** 2016-01-08

**Authors:** Pengfei Tian, Kresten Lindorff-Larsen, Wouter Boomsma, Mogens Høgh Jensen, Daniel Erik Otzen

**Affiliations:** 1 Niels Bohr Institute, University of Copenhagen, Blegdamsvej 17, 2100, Copenhagen, Denmark; 2 Linderstrøm-Lang Centre for Protein Science and Structural Biology and NMR Laboratory, Department of Biology, University of Copenhagen, Ole Maaløes Vej 5, DK-2200, Copenhagen N, Denmark; 3 Interdisciplinary Nanoscience Center (iNANO), Centre for Insoluble Protein Structures (inSPIN), Department of Molecular Biology, Aarhus University, Gustav Wieds Vej 10, 8000, Aarhus C, Denmark; University of Akron, UNITED STATES

## Abstract

In addition to their well-known roles in neurodegenerative diseases and amyloidoses, amyloid structures also assume important functional roles in the cell. Although functional amyloid shares many physiochemical properties with its pathogenic counterpart, it is evolutionarily optimized to avoid cytotoxicity. This makes it an interesting study case for aggregation phenomenon in general. One of the most well-known examples of a functional amyloid, *E*. *coli* curli, is an essential component in the formation of bacterial biofilm, and is primarily formed by aggregates of the protein CsgA. Previous studies have shown that the minor sequence variations observed in the five different subrepeats (R1-R5), which comprise the CsgA primary sequence, have a substantial influence on their individual aggregation propensities. Using a recently described diffusion-optimized enhanced sampling approach for Monte Carlo simulations, we here investigate the equilibrium properties of the monomeric and dimeric states of these subrepeats, to probe whether structural properties observed in these early stage oligomers are decisive for the characteristics of the resulting aggregate. We show that the dimerization propensities of these peptides have strong correlations with their propensity for amyloid formation, and provide structural insights into the inter- and intramolecular contacts that appear to be essential in this process.

## Introduction

While protein amyloid has typically been associated with pathological conditions such as amyloidosis and neurodegenerative diseases including Parkinson’s, Alzheimer’s and Huntington’s disease [1, 2], it is increasingly clear that amyloid can also be a benign state with various important functional roles in their hosts [3–5]. Recent advances reveal that these functional amyloids share many physical and chemical properties with the pathological variants [6–8]. For instance, curli, natural amyloid fibers that are involved in biofilm formation by *E*.*coli*, can induce inflammatory responses of host tissue, similar to that known from the Alzheimer associated Aβ [9]. Moreover, a molecule that stimulates the oligomer assembly of α-synuclein has been demonstrated to have similar effect on the curli amyloid protein CsgA [10]. Despite their similarities, the properties of pathogenic and functional amyloid diverge in one important aspect: functional amyloid is regulated by the organism to avoid the cytotoxicity associated with the formation of their pathogenic counterparts [11–13]. This renders functional amyloid highly important as model systems for understanding amyloid growth.

**Fig 1:**
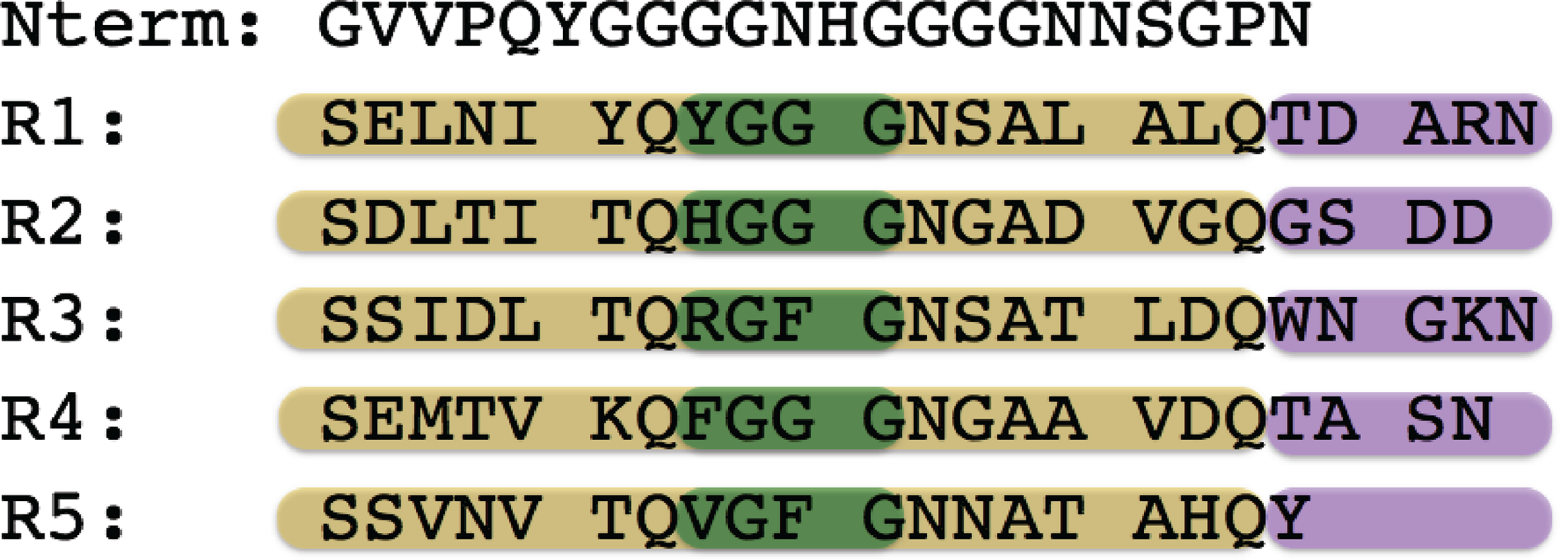
CsgA primary sequence including sub-repeat (R1 –R5) with the alignment of internally conserved Ser-X5-Gln-X-Gly-X-Gly-Asn-X-Ala-X3-Gln-X residues.

Curli is one of the best-understood functional amyloid system [14]. It is mainly formed by aggregation of the subunit protein, CsgA (Fig 1). CsgA is a soluble protein secreted through the outer membrane by protein CsgG, nucleated by the CsgB and assembled into amyloid fibrils on the surface of the cell [15, 16]. Recently, progress has been made on the characterization of the detailed amyloid protein structure by solid state NMR [6, 17], and amino acid proximity information extracted from residue-residue coevolution has revealed that CsgA forms a β-helical structure inside the amyloid fibrils [18]. However, the details of the folding pathway of CsgA and the assembly pathway remain unclear.

The amyloid core of CsgA contains five repeating units, R1-R5, each consisting of 19–23 amino acids (Fig 1). Each of these repeats is found to form a single turn in the final β-helical state [18]. A recent set of studies probed these subunits independently, demonstrating that the nucleation and polymerization of CsgA is encoded in the minor variations in amino acid composition of these different subunits [19–21]. For example, deletion of R1 or R5 prevents CsgA amyloid formation, while R2 can be omitted without affecting the overall aggregation process. Also, peptides corresponding to R1 and R5 were shown to aggregate efficiently by themselves, while R2 lacked this property. Likewise, studies have examined the effect of the amino acid composition of the turn region separating the repeats, showing similarly strong dependencies [22]. These detailed experimental characterizations of the effects of minor sequence variations on global aggregation properties provide us with a unique opportunity to investigate the link between early oligomeric structure and the mature fibrillar amyloid state. One of the main difficulties in understanding the process of amyloid formation at a molecular level is the range of scales involved—both in time and size, a significant effort has been made towards the study of this problem using computer simulations recently [23–27]. Initially, monomers are thought to associate into oligomers or protofibrils, which will eventually, under specific conditions, assemble into full fibrils. The initial dimerization is known to be critical for oligomerization and subsequent fibril growth [25, 28, 29], and it is also considered as a key primary event of the pathogenesis of for instance prion diseases [30], but it is still largely unknown to which extent the structural properties of the dimer are linked to that of the fibril [31]. The small size of the CsgA peptides, combined with the evolutionary optimization of the aggregation propensities, makes them attractive model systems to test the early events in protein aggregation in silico.

In the present study, we performed atomic-level computer simulations of the monomer-monomer interactions of the individual CsgA subrepeats which were exprimentally characterized in the studies described above, and investigate whether the effects we observe in their dimerization translate directly to the experimentally observed aggregation properties. Using a recently developed Monte Carlo (MC) enhanced sampling technique, we obtain a converged thermodynamic characterization of the free energy landscape of the interacting monomers, and use this to quantify the degree to which different inter- and intramolecular contacts are formed. Our study shows a remarkable correspondence between the propensities for dimer formation and the CsgA aggregation propensities previously reported, suggesting that formation of specific contacts already at the dimerization stage can dramatically influence the amyloid pathway.

## Results

### Characterization of monomer conformations

**Fig 2:**
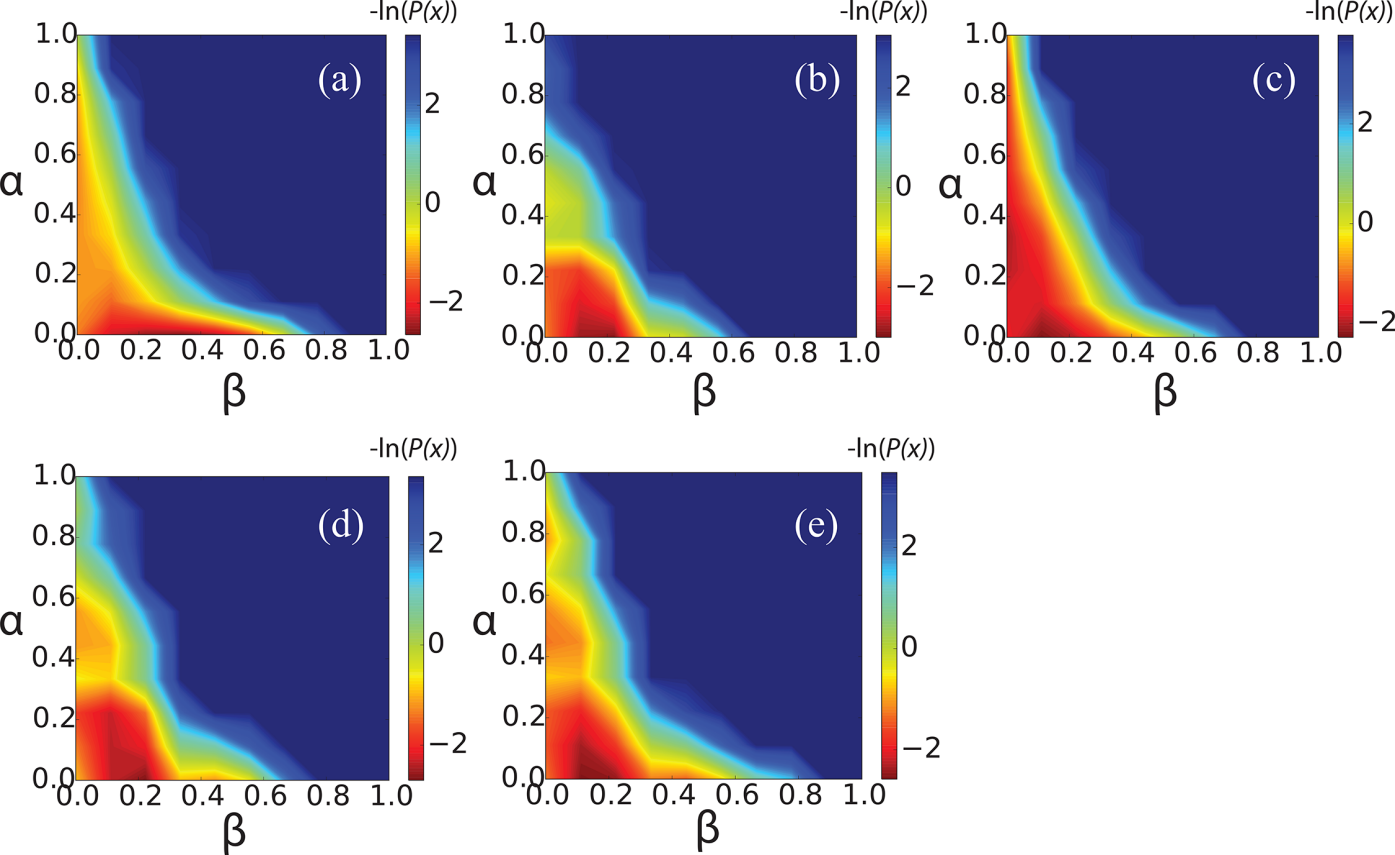
Free energy *F*(α,β) at 300K of the monomer of R1—R5((a)—(e)), calculated as a function of the α-helix content (Y-axis) and β-strand content (X-axis). *F*(α,β) = -ln(*P(x)*)), where *P(x)* represents the probability of state *x*.

The subrepeats of the CsgA protein generally have high sequence similarity (Fig 1), but display variations that have been demonstrated to have a dramatic impact on their propensity to aggregate[19–21]. This observation raises the question whether the minor variations in primary sequence encode critical change in secondary structure, which might be observed already at the monomer level. To investigate this question, we began by characterizing the conformational ensemble of the monomeric state of the individual R1-R5 repeats, by determining the free energy, *F*(α, β), calculated as a function of α-helix and β-strand content (Fig 2).

We conducted our simulations using an all atom force field with an empirical implicit solvent model, PROFASI [32, 33]. This simulation framework has been successfully used to characterize thermodynamics of protein folding, as well as native structure ensembles with experimental data restraints [34–37]. Recently, combining with Monte Carlo method, it has been used to study the dimerization and aggregation of Aβ_42_ [38, 39] and Aβ_16–22_ peptides [40], the misfolding of α-synuclein [41] and Copper, zinc superoxide dismutase 1 [42], the simulation results agree very well with the experimental studies on the kinetic and equilibrium properties. As an additional test of the forcefield, we conducted a series of simulations on the (AAQAA)_3_ 15-mer peptide, a system which has previously been used to test molecular forcefields. These results show agreement with experiment as good as optimized all-atom MD potentials [43] (Figure A in S1 File) To explore a wide range of conformations more efficiently, in our current study, sampling was performed in a multicanonical (flat histogram) ensemble and subsequently reweighted to obtain Boltzmann statistics at 300K (see Methods). The secondary structure content was calculated based on Ramachandran angles for each residue using STRIDE [44].

Unlike what has previously been observed for monomeric Aβ peptides [25, 38, 45] and α-synuclein [41, 46, 47] which all have strong propensity to form β-strands, we find that the averaged ensembles have α-helix content < 10% and β-strand content < 20% (Fig 2 and Figure B in S1 File). The structural diversities of the monomers are described with more details by calculating the residue contact probability map of the whole ensemble at room temperature (Figure C in S1 File). Our results thus indicate that the R1–R5 monomer is not well structured in its free state, some β-hairpin like residue-residue contacts are formed with very low probabilities at 300K. One potential explanation could be that monomeric disorder is required to maintain solubility and avoid early aggregation of CsgA before it is secreted to the cell surface [16]. The generally low levels of secondary structure make it difficult to differentiate between properties of the individual subrepeats. Consequently, at the monomer level, we find little correlation between simulated secondary structure propensities and the experimentally observed aggregation propensities [19].

### Dimer simulations

#### Peptides binding free energy

Given their disordered nature, the monomer simulations provide little information on the aggregated state of CsgA. We therefore proceed with a simulation analysis of dimer-formation for each of the subrepeats. It is known to be computationally challenging to converge dimer simulations. We initiated our studies using a standard parallel tempering (replica exchange) setup, but failed to obtain reliable free energy estimates (see Methods) and were therefore forced to abandon this approach. To overcome these problems we adopted a generalized ensemble technique that aims at optimizing the number of transitions from high to low energy (in this case between monomeric and dimeric species), using the energy-dependent diffusion profile (see Methods). Compared to the parallel tempering method, the sampling speed was improved by 30% to 460% (depending on the subrepeat) in terms of round trip time, thereby accumulating more statistics especially around the transition states (see Methods). The simulations of all the systems are fully converged as we can see from the error analysis of the thermodynamic properties using the Jackknife method in the Figure D in S1 File. In each simulation, we initialize with random extended structures in a cubic box with side lengths of 100Å, corresponding to a concentration of around 4.8 mg/ml.

**Fig 3:**
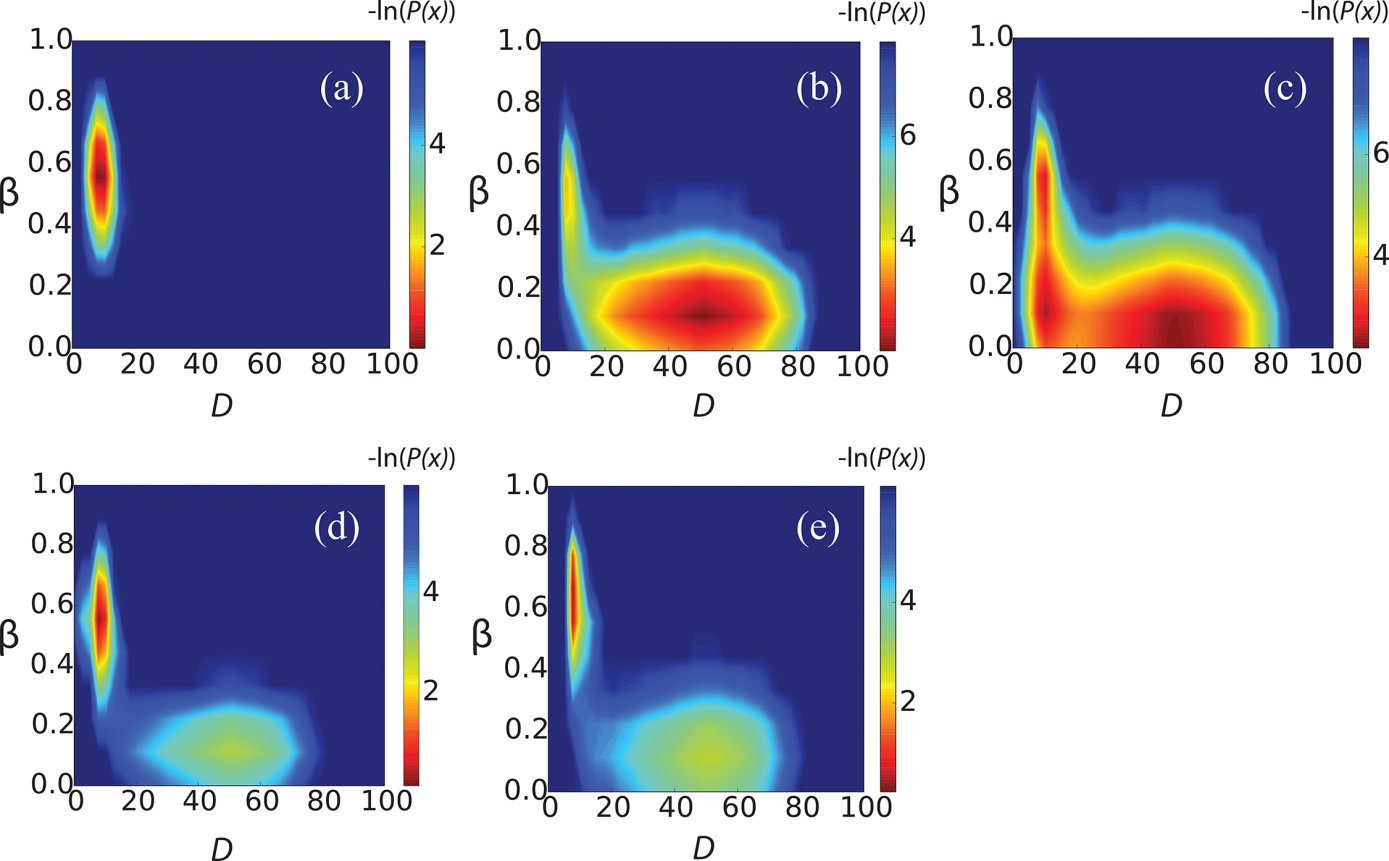
Free energy of binding (-ln(*P(x)*)) for R1 –R5 dimer at the 300K. *D* (X-axis) is the distance of the center of the mass between the two-peptide chains and β content (Y-axis) is the percentage of β-strand within the structure. (a)–(e) correspond to R1 –R5, respectively.

To investigate the character of the transition between phases of the dimerized and separated chains, we constructed equilibrium free energy surfaces at 300K (Fig 3). Despite very high sequence identity, the five peptides display dramatically different dimerization tendencies. For R1, R4 and R5, the global free energy minimum at high β-strand content and short distance of the two chains indicates that dimeric β-hairpin states dominate the population. In contrast, the R2 peptides mainly stay in the unbounded disordered states, while R3 shows an intermediate binding affinity, marginally stronger than R2. Compared to the monomers, which are primarily disordered, the ensemble of dimer conformations contains significantly larger β-hairpin population, especially for R1, R3, R4 and R5. We thus see a remarkable stabilizing effect on the hairpin induced by the formation of dimer. The calculated standard binding free energy (Δ*G*) are -4.7 kcal/mol, -2.9 kcal/mol, -4.3 kcal/mol, -6.0 kcal/mol and -8.3 kcal/mol for R1-R5 respectively. Δ*G* is calculated as:

$$\Delta G=-k_{B}\mathrm{Tln}\left(\frac{K_{a}}{V_{\mathrm{ref}}}\right),$$

where ***K***_***a***_ is the association constant, **k**_***B***_ is the Boltzmann constant, ***T*** is the room temperature (300K), and ***V***_***ref***_ is the association constant and V_ref_ is the reference volume in units consistent with the units of concentration in Ka (mol/L). Ka = [AB]/[A][B], where A represents peptide chain A and B represents chain B. Both A and B start out as monomers with an initial concentration of 1 per simulation box (100Å in length, i.e. 10^−24^ m^3^ or 10^−27^ L), corresponding to 1/600 mol/L.

#### Aggregation propensity

**Fig 4:**
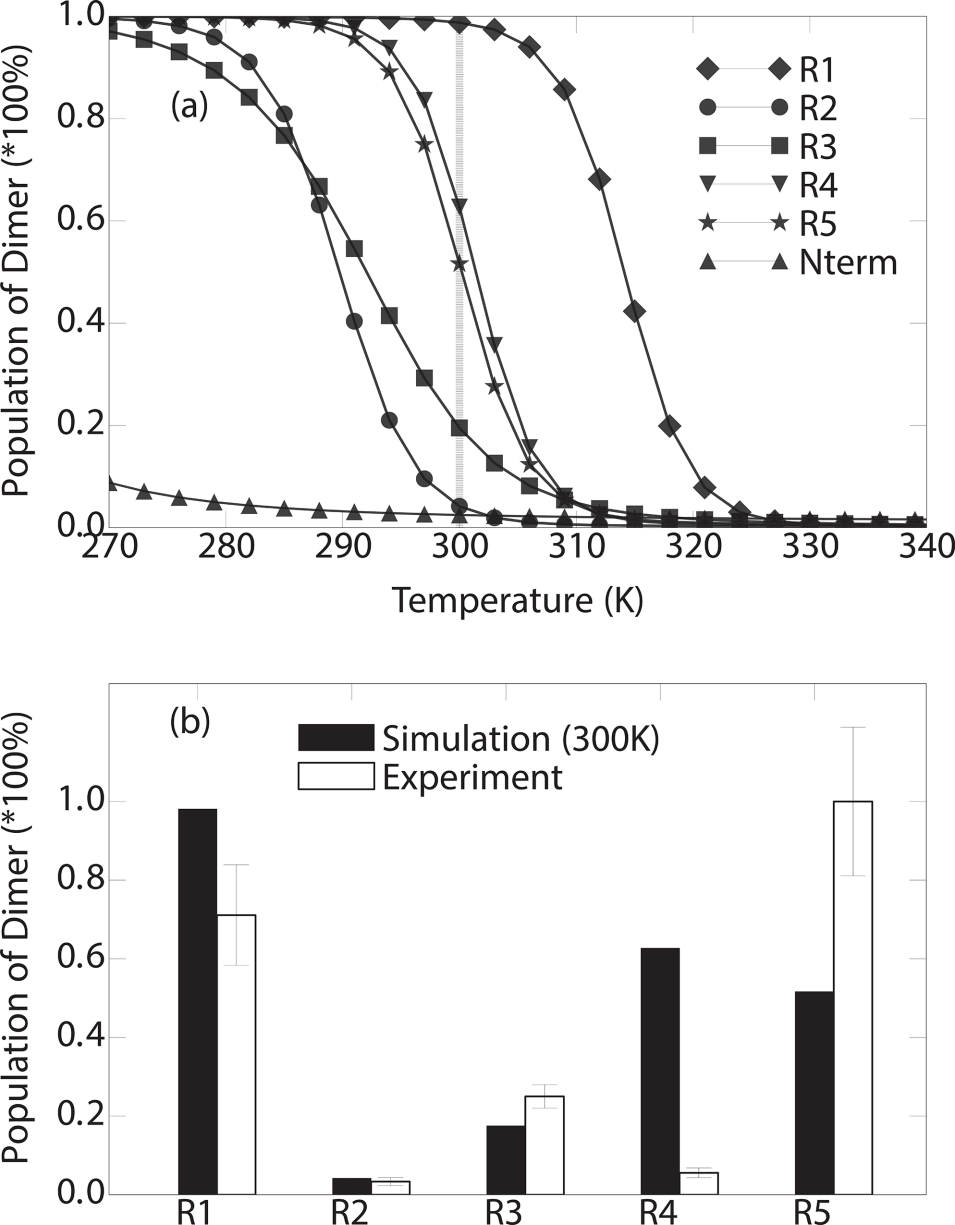
(a) Temperature dependent population of the dimer phase. (b) Dimerization propensity obtained by the simulation at 300K compared to the experimental measurement of the self-aggregation propensity at room temperature (*i*.*e*. around 300K)[19].

A central question is how strongly the observed dimer characteristics are linked to the properties of the fully formed aggregate state. Fortunately, aggregation of CsgA subrepeats has been extensively studied experimentally [19–21], showing substantial differences between the repeats. We proceed with a more detailed comparison to these experimental data by calculating melting curves from the temperature dependent dimerization simulations described in the previous section (Fig 4A). We observe that the melting temperatures for R1, R4 and R5 (312K, 300K and 300K, respectively) are clearly higher than those for R2 and R3 (291K and 293K, respectively), pointing toward a considerable difference in stability between the two groups.

The experimental data takes the form of aggregation propensities for the repeats measured using thioflavin T fluorescence [19, 21]. The simulation results projected to the distance of the center of the mass between the peptide chains (*D*) show strongly bimodal distribution at the room temperature (Figure E in S1 File), the aggregated phase and the detached phase are separated by a clear free energy barrier at *D* = 15Å. Therefore, to allow for comparisons to our simulated data, we define the proteins to be in a dimeric state when the *D* is below 15Å. The percentage of dimer population observed in simulation is shown in Fig 4. After normalizing the populations obtained from both the simulation and the experimental data, we observe that for 4 out of 5 of the subrepeats: R1, R2, R3 and R5, there are very strong correlations between the dimerization propensity obtained by the simulation and the oligomerization propensity obtained by experiments (Fig 4). To further validate the robustness of our conclusion, we reproduced our analyses using a cutoff of *D* = 10Å, and show that the aggregation profile is consistent with the D = 15Å result (Figure F in S1 File). Furthermore, to rule out disordered states within the cutoff, we have added an additional analysis using β-strand content as a reaction coordinate, calculating the temperature dependent population with D < 15 Å and β-strand-content > 50% (Figure F in S1 File). The relative aggregation propensities of different systems (except for R4) were found to remain consistent with the experimental data at room temperature.

As a control simulation, we include a dimerization study of the 22 amino acids long N-terminal peptide from CsgA that is not involved in curli fibril formation (Nterm in Fig 1). The simulations reveal that this peptide does not substantially dimerize at any temperature within a broad temperature range. This is consistent with previous findings that this part of the sequence is not involved in the amyloid core of the curli structure [20, 21].

The observed correlation between dimerization propensities in simulation and the experimentally observed rate of aggregation for these subrepeats suggests that structural properties of the full fibril are to some extent encoded in the dimer structure. This can be seen as providing some support to previous studies on neurodegenerative-associated amyloid proteins, where dimerization has been reported as the primary trigger for oligomerization and further aggregation [29, 30, 48]. The R4 subrepeat, however, stands out with a significant discrepancy between simulation and experiment. This prompted us to further investigate the detailed structural conformations and contacts formed during the dimerization process for the different repeats.

#### Intra- and inter-chain contacts

**Fig 5:**
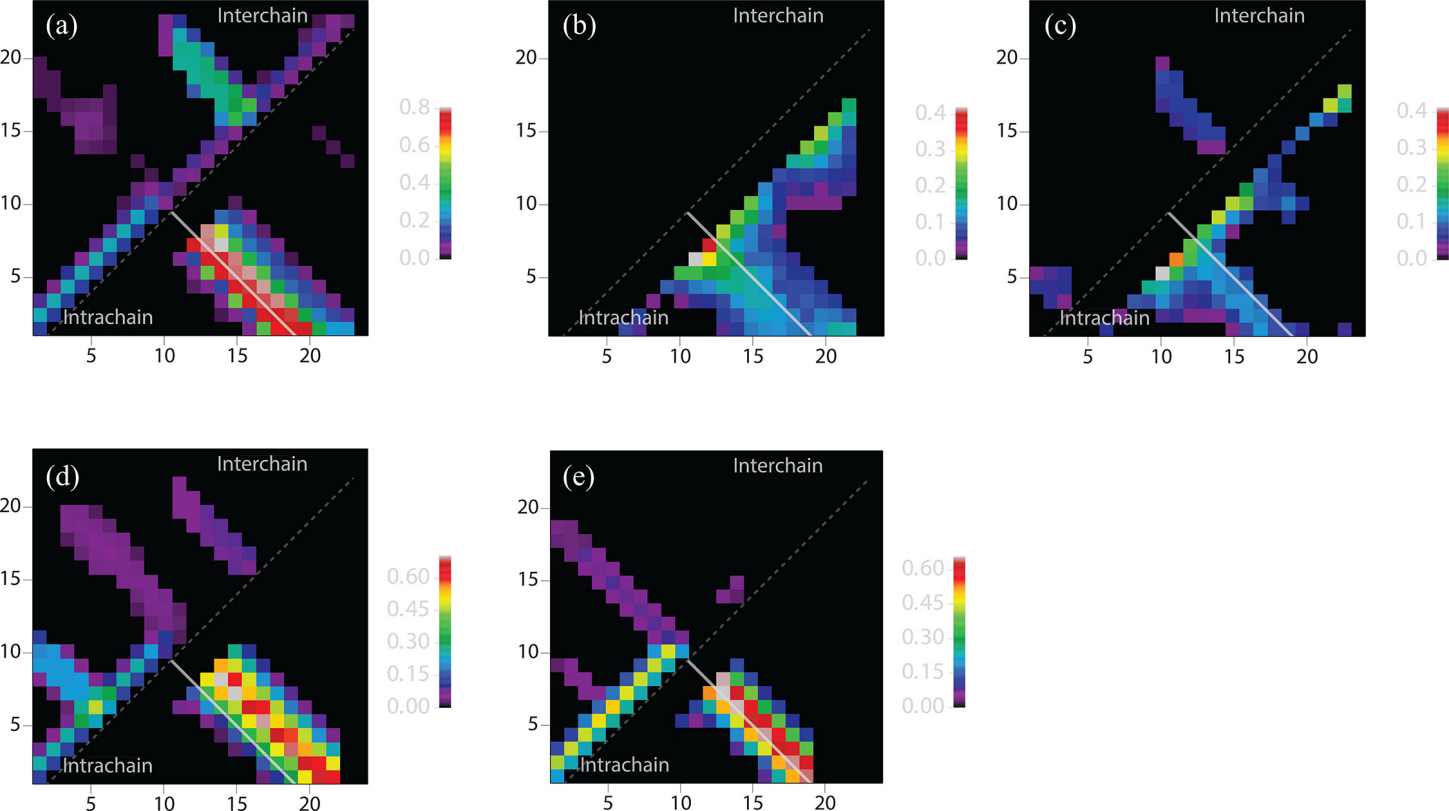
Residue contact probability map for contacts within and between the two chains at 300K. Intrachain and interchain pair contact probabilities are shown below and above the main diagonal (dashed white line), respectively. The solid white shows the diagonal of a 19×19 matrix in each plot. (a)–(e) correspond to R1 –R5, respectively.

**Fig 6:**
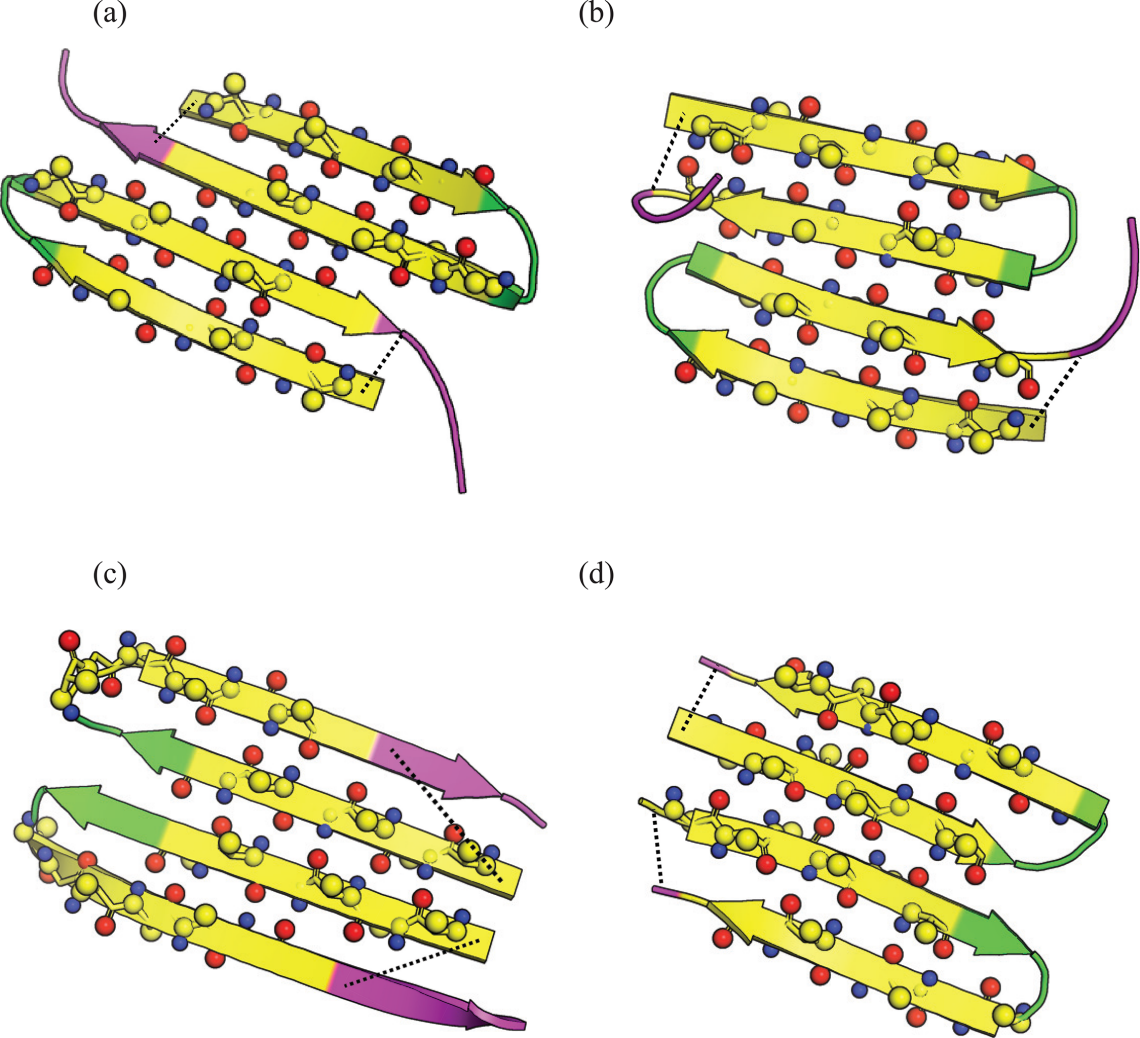
Representative dimer state of R1 (a), R3 (b), R4 (c) and R5 (d). Residue index of 8 to 11 are colored in green and residue 19 to the end of the chain is colored in purple as Fig 1. For residues 2 to 7 and 12 to 17, hydrogen-bonded backbone oxygens and protons are illustrated in red and blue, respectively. And the Cβ atoms on the side chains are shown as well. The dashed line is the distance between CA atoms of residues 1 and 19. A more detailed version of these structures is shown in the Figure G in S1 File.

For a more detailed description of the tertiary organization of the dimers, we calculated the probability of each possible intrachain and interchain contact based on the whole ensemble for each system. Contact maps were constructed based on residue pairs with *CA*-*CA* distance below 8Å, containing the percentage of time that this contact is formed during a simulation (Fig 5). The parallel and anti-parallel pattern in the contact map demonstrates that dimer states occur with different orientations. R2 is an exception, for which almost no binding contacts between the two chains are observed, which agrees with the low aggregation propensity observed in experiment [19]. To support the visual interpretation of the contact maps, we also isolate representative structures for each subrepeat as cluster centroids (using the Gromacs g_cluster program [49]) (Fig 6).

Different dimer conformations can be classified and labeled according to the inner two strands of the dimer (whether the β-strand sequence is closer to either the N-terminus or the C terminus, and whether the two inner strands are either parallel or antiparallel): NN-parallel, NC-parallel, CC-parallel, NN-antiparallel, NC-antiparallel, and CC-antiparallel. From Fig 5 we can see the most populated dimer state for R1 and R3 is CC-antiparallel (Fig 6A and 6B). Moreover, R1 can also form an NN-parallel dimer. R4 forms all types of different dimers, ordered in probability from high to low: NN-parallel, NN-antiparallel, and NC-antiparallel and CC-antiparallel. The dimers of R5 include NN-parallel, NN-antiparallel, and NC-antiparallel. In contrast to the dimer of Aβ which is stabilized mainly by the hydrophobic effect [39, 50], the primary force driving the dimer formation is the inter and intra chain hydrogen bonding. Presumably, the oligomer could potentially be stabilized further with the formation of a hydrophobic core between opposite β sheets, similar to the model of the CsgA monomer [16, 18] or other peptide fibrils [51], when more chains are involved.

The R1 to R5 subrepeats are known to behave differently in their ability to polymerize with CsgA seeds [21]. In contrast to R1, R3, and R5, which polymerize rapidly in contact with a CsgA seed, the R2 and R4 subrepeats have been reported to remain unpolymerized for 48 hours at room temperature [20]. For R2, this result is entirely consistent with our simulations, which demonstrate that this subrepeat does not form stable interchain hydrogen bonds (Figs 3 and 5). In contrast, R4 readily dimerizes in our simulations. However, R4 exhibits several particular characteristics compared to the other repeats. While R4 shares characteristics of the inter-chain contacts with R1, R3 and R5, there are marked differences in the intra-chain contacts: the hairpin turn of R4 is at residue position 10–13 while R1, R3, R5 are located at around residue 8–11 (Figs 5D and 6C). Interestingly, this latter position is identical to the location of the turns in the full-length CsgA amyloid state [16, 18]. The aggregation mode has previously been reported to be highly related to the arrangement of the sequence within the repeat [22], suggesting that the lack of CsgA-seeded R4 polymerization could be explained by this incompatibility in topology.

## Conclusion

Accurate descriptions of the multiple length- and time scales involved in protein aggregation remains an important challenge in molecular simulation [52–54]. In this study, we investigated whether characteristic properties observed in the early oligomerization steps are reflected in the final aggregate state for the different subrepeats in the functional amyloid protein CsgA. By combining a minimalistic and highly efficient force field with a state-of-the art Monte Carlo simulation methodology, we have successfully converged the free energy landscapes of both the monomer and dimer states. The monomers were found to be primarily disordered. However, the dimerization process induces more well-defined structure, leading to distinct topologies for the different subrepeats. In our simulations, hydrogen bonding seems to be the main stabilizing force of dimer formation, in line with previous findings from both experiments and simulations [55–57]. We found the peptide repeats R1, R3, and R5, which have the highest dimerization propensities, to form similar hairpins with the X-G-X-G amino acid motif at the turn position. Finally, we calculated the dimerization propensity for each subrepeat and found remarkable agreement with both the experimentally measured propensities for self-polymerization [19] and for polymerization with CsgA fiber seeds [21]. While strong correlations were observed over the propensities of the R1, R2, R3 and R5 subrepeats, R4 displays substantial discrepancies. Experimentally, R4 was previously reported to stand out for its inability to polymerize even in the presence of CsgA seeds. The contact maps obtained from our simulations suggest that the reason for this incompatibility could be the shift in turn position compared to the other subrepeats. Based on the amyloid state of the CsgA monomer we determined in the previous study [18], the hydrogen bonding seems to be the main stabilizing force between the neighboring subrepeat. And the conserved pattern of hydrogen bonded residue “ladders” are contributed by the residues of index range 1–7 (the first β-strand) and 12 to the end of the subrepeat (the second β-strand) with the neighbouring subrepeat at the same residue positions. The residue index here is numbered on each subrepeat. For instance, the 14^th^ residue ALA on each subrepeat form hydrogen bonds with the ALA14 from the neighboring subrepeat. Given the aggregated model we obtained from the simulation as shown in the Fig 6 and the Figure G in S1 File, for R1, R3 and R5, either the first or the second β-strand could “stack” onto the CsgA fibril with the conserved hydrogen bonding pattern. However, as to R4, the residue 12–14 is in the turn position instead of β–strand as the other subrepeats, it could potentially prevent the formation of hydrogen bonds with the same pattern.

A natural next step would be to extend our simulations to three chains, to evaluate whether the trimeric states follow the same pattern, and perhaps hint at the assembly into the full aggregated state. We have initiated work in this direction, but have so far been unsuccessful in obtaining converged estimates using an unbiased simulation approach. Even attempts at restricting two copies to a fully formed dimeric state to probe the association of the third failed to provide conclusive results. Incorporating bias terms from experimental data to drive the system between states of interest might be a fruitful path forward in this respect [58, 59]. Overall, our results indicate, however, that even an approach limited to two-chain simulations, when fully converged, can potentially provide valuable insights into the early stages of amyloidgenesis, by quantifying the formation of essential contacts that drive the aggregation process. In particular, such contact information is an important complement to the site-specific aggregation propensities that can be obtained using more standard sequence-based aggregation prediction algorithms.

## Methods

All simulations were conducted using the PROFASI force field [32]. This implicit solvent force field has previously been used to characterize the folding and refolding thermodynamic properties of various proteins, and has succesfully been applied to aggregation studies [32]. The overall energy potential is composed of four terms:

$$E=E_{\mathrm{loc}}+E_{\mathrm{ev}}+E_{\mathrm{hb}}+E_{\mathrm{sc}}\;,$$ (1)

where *E*_*loc*_ represents the local interactions between atoms connected by only a few covalent bonds, while *E*_*ev*_, *E*_*hb*_ and *E*_*sc*_ take into account the non-local character. *E*_*ev*_ is the energy term of exclude volume effect. *E*_*hb*_ is the hydrogen bond potential and *E*_*sc*_ represents charge-charge interaction between sidechains. For further details, we refer to the original description of the model [32, 60].

The software framework PROFASI [33] is used to carry out the Monte Carlo (MC) simulation, using five different kinds of conformational updates: the pivot move, biased Gaussian steps (semi-local move) [61], side chain move, rotation move and translation move. Default settings are used for the weights of the updates. In PROFASI, by design, the bond lengths of covalent bonds and the bond angle between adjacent covalent bonds are kept fixed, leaving torsion angles in the backbone and side chains as the only degrees of freedom.

The monomer simulations were run using a standard Metropolis-Hastings Monte Carlo setup at 300K. For the dimer simulations we experienced severe convergence problems and experimented with various enhanced sampling algorithms in our efforts to obtain reliable free energy estimates. One method that turned out to be particularly well suited for this problem is a recently developed generalized ensemble technique based on flattening the energy-specific diffusion profile [62, 63]. Below, we briefly introduce the method and compare its performance to the commonly used parallel tempering (replica exchange) technique.

### Diffusion-optimized ensembles

In generalized ensemble techniques, the standard Boltzmann expression is replaced with a weight function *w*(*E*). Various proposals for choices of efficient weight functions have been presented in the literature [64–66]. One of the most common strategies is the multicanonical form:

w(E)=1/g(E), (2)

where *g*(*E*) is the density of state. Thus, the energy histogram converges to a flat distribution. However, a flat histogram is not the optimal choice if one wishes to minimize the first passage time (*τ*) between the minimum and maximum energies (*E*_*min*_ and *E*_*max*_) [63]. If we assume that the dynamics can be described as one-dimensional diffusion along the reaction coordinate (in our case, along the energy *E*), then the mean first-passage time *τ* for “round trips” that cross the whole energy space is

$$\tau =\int_{E_{\min}}^{E_{\max}}\frac{1}{D(E)P(E)}dE,$$ (3)

where *D*(*E*) is the position-dependent diffusion coefficient along the reaction coordinate *E* and *P*(*E*) is the probability distribution of energy.

Eq 3 obtains its minimum when

$$P_{\mathrm{opt}}\left(E\right)=\frac{1}{\sqrt{D\left(E\right)}},$$ (4)

with

$$D\left(E\right)=\frac{\pi }{\Delta t}\frac{Z_{C}{\left(E\right)}^{2}}{Z_{H}{\left(E\right)}^{2}},$$ (5)

in which Δ*t* is the sample interval, *Z*_*H*_(*E*) is the ordinary energy histogram and *Z*_*C*_(*E*) is the transition histogram. Specifically, given the simulation trajectory of *E*_*i*_ = *E*(*i*Δ*t*) (*i* = 1,2 …), *Z*_*H*_(*E*) = *N*/Δ*E* where *N* is the total number of samples (*E*_*i*_) that drop into the same bin of the width ΔE, *Z*_*C*_(*E*) is the number of transitions that cross a specific energy “cut”:

$$Z_{C}\left(E\right)=1/2\;\sum_{i}\Theta \left[\left(E\left(i\Delta t\right)-\;E\right)\left(E-E\left(i\Delta t+\Delta t\right)\right)\right],$$ (6)

where Θ is the Heaviside step function. In practice, the optimized weights are estimated through a feedback iteration,

$$\ln\;w^{n}(E)=\ln w^{n-1}(E)-\ln Z_{C}(E),$$ (7)

where w^*n*−1^ and w^*n*^ are the old and new weights respectively. After this, the Boltzmann statistics can be recovered using reweighting techniques [67]. The details of calculating the free energy are introduced in the previous study [68]. All the monomer and dimer simulations we carried out were started from fully extended structures.

**Fig 7:**
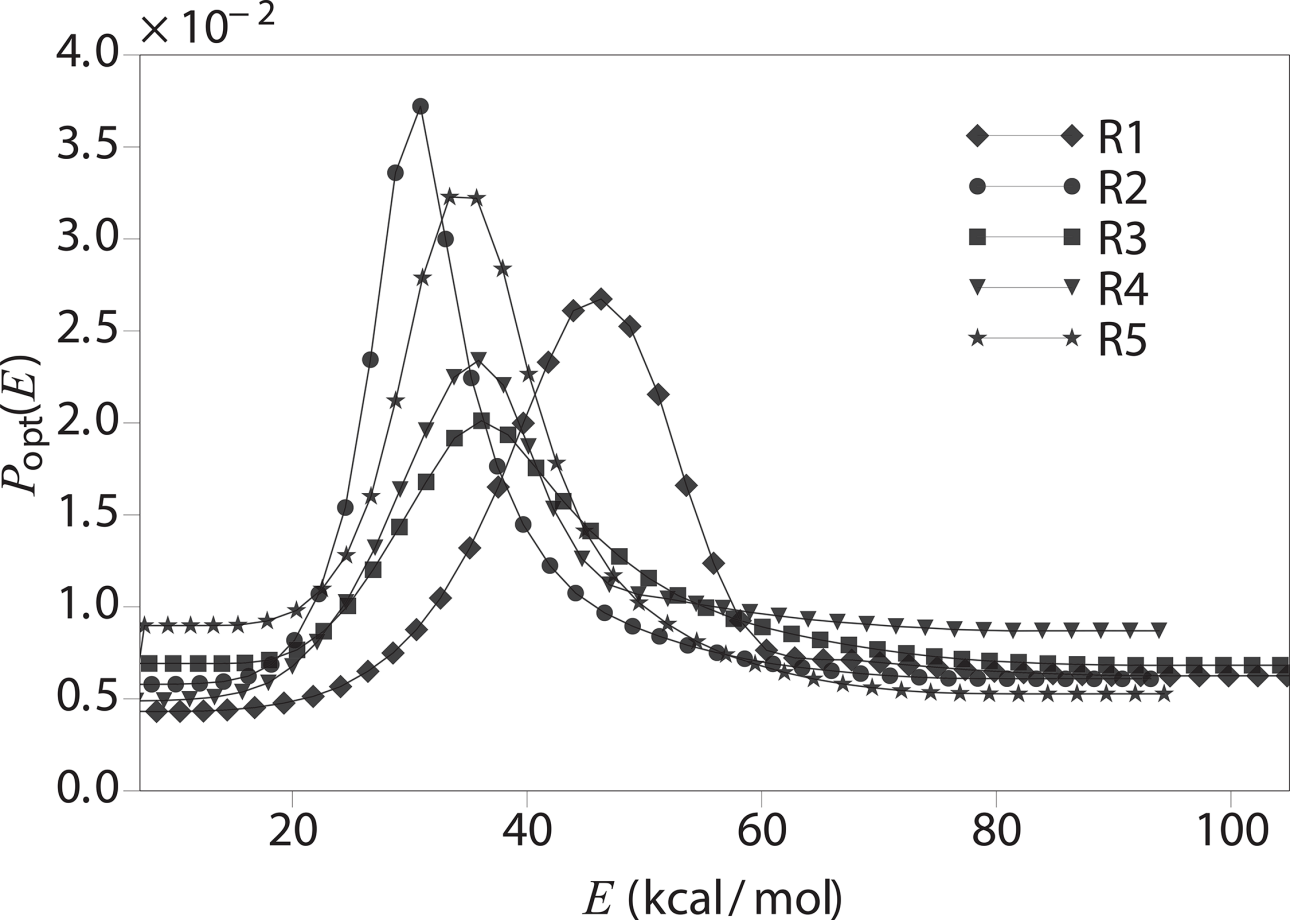
Energy distribution of R1-R5 after the feedback (Eq 7).

In our simulation of dimers, the initial runs of 1 × 10^8^ MC sweeps (one sweep is equal to the number of degrees of freedom in the system) of each system are carried out to generate the optimized weight by the Eq 7. The diffusion-optimized distributions are illustrated in Fig 7.

**Fig 8:**
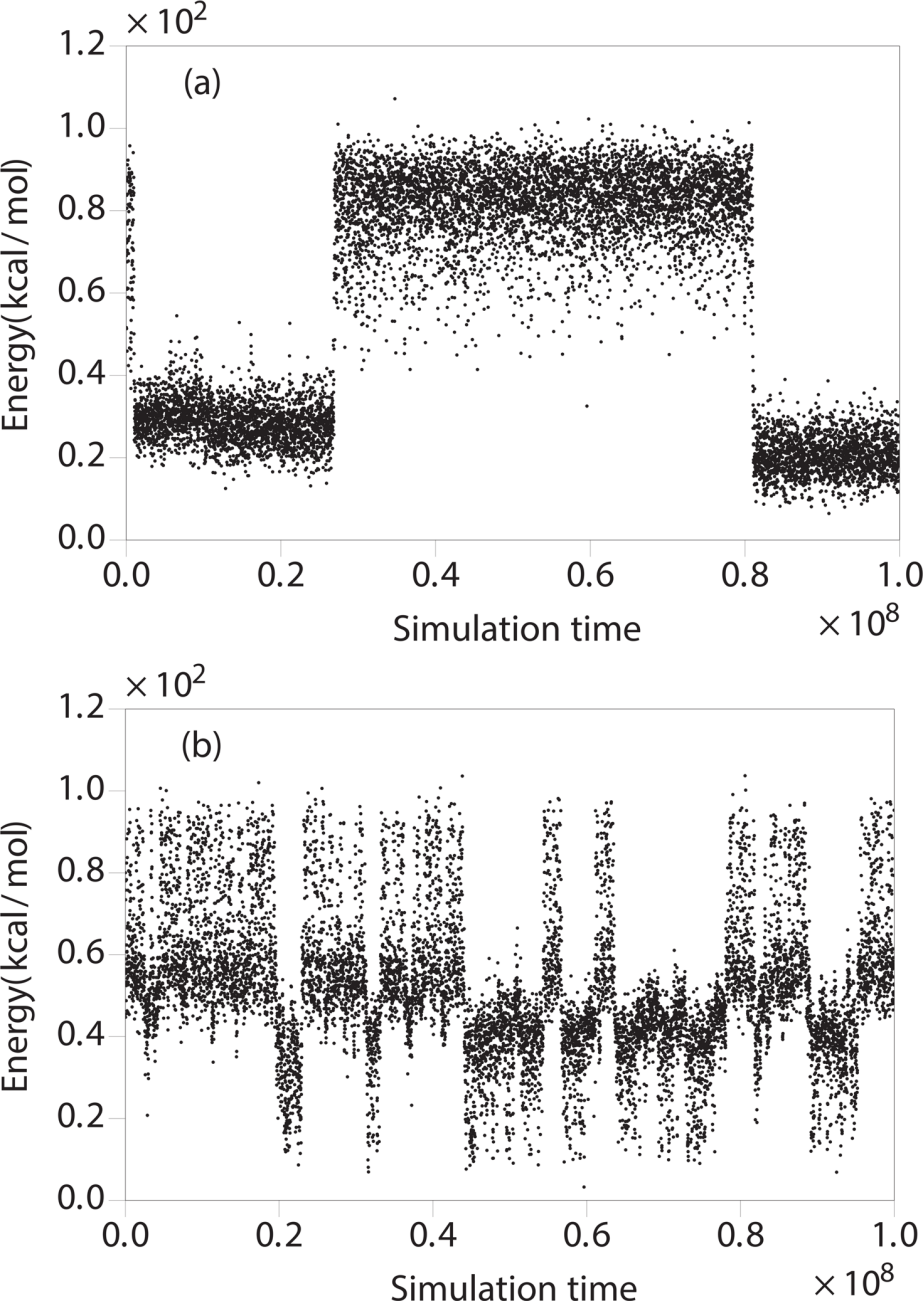
(a) Representative simulation trajectories (of R1 dimer) of energy by the PT method and by (b) the diffusion-optimized ensemble method.

Using the optimized weights for the simulation, we observe a dramatic speedup of sampling in terms of the number of tunneling events within the same simulation time (Fig 8, Table 1). Compared to the parallel tempering (PT) technique, the diffusion-optimized ensemble approach consistently gains speedup from 30% to 460% for different subrepeats. Both kinds of the simulations are carried on 16 parallel replicas within the same temperature range 279−367 K. In our setup, the PT method uses the default settings of the software package PROFASI [33], where the 16 temperatures are chosen according to a geometric distribution in the temperature range 279−367 K. We note that it has been shown that the efficiency of PT can be optimized considerably using diffusion considerations similar to those described above [69]. In our case, the diffusion optimized ensemble is carried out in a generalized ensemble framework, using the MUNINN [68, 70] software package.

**Table 1:** Round-trip speed comparison.

Repeat	Num of tunneling events (PT)	Num of tunneling events (Diffusion Optimized)
R1	9	50
R2	19	65
R3	55	82
R4	14	54
R5	17	22

## Supporting Information

S1 File Comparison of secondary structure propensities between calculation and experiment. We show the temperature dependent helical fraction of the (AAQAA)_3_ peptide obtained from previous experiment and simulations using PROFASI force field (Figure A). Free energy of R1 **–**R5 monomer at the 300K projected to the reaction coordinates of the β-strand content (beta) and the α-helix content (alpha) (Figure B). Residue contact probability map for R1–R5 (a-e) monomer at 300K. The solid white shows the diagonal of a 19×19 matrix in each plot. The minimum residue distance is 5. Contact maps were constructed based on residue pairs with CA-CA distance below 8Å, containing the percentage of time that this contact is formed during a simulation (Figure C). Mean and error estimated for the temperature dependent population of the dimer phase (main text Fig 4). Since we have 8 independent simulations for each system, the mean and error here are estimated with the Jackknife method by excluding samples from one simulation at a time (Figure D). Free energy of binding for R1 **–**R5 dimer at the 300K(main text Fig 3) projected to the reaction coordinate *D* (a) and β-strand content (b). The dashed lines in (a) correspond to D **=** 10 Å and 15 Å. The dashed line in (b) corresponds to beta (β-strand content) **=** 0.5 (Figure E). Temperature dependent population of the dimer phase with the D < 10Å(a) and Temperature dependent population of the dimer phase with D < 15Å and β-strand content > 50%(b) (Figure F). The detailed version of the dimer states of R1 (a), R3 (b), R4 (c) and R5 (d) in the main text Fig 6. Nitrogen donor and oxygen acceptor involved in the backbone hydrogen-bonding are illustrated in blue and red, respectively. The intra- and inter- chain hydrogen bonds are shown as yellow dashed line. All the sidechain atoms are not displayed here. The models are drawn with PyMOL (Figure G).
(DOCX)
