# Supplementary material for: A Monte Carlo Study of the Early Steps of Functional Amyloid Formation
Source: PLoS One. 2016 Jan 8;11(1):e0146096. doi: 10.1371/journal.pone.0146096 (PMC4706413; doi:10.1371/journal.pone.0146096)
Supplement: S1 File — We show the temperature dependent helical fraction of the (AAQAA)3 peptide obtained from previous experiment and simulations using PROFASI force field (Figure A). Free energy of R1 –R5 monomer at the 300K projected to the reaction coordinates of the β-strand content (beta) and the α-helix content (alpha) (Figure B). Residue contact probability map for R1–R5 (a-e) monomer at 300K. The solid white shows the diagonal of a 19×19 matrix in each plot. The minimum residue distance is 5. Contact maps were constructed based on residue pairs with CA-CA distance below 8Å, containing the percentage of time that this contact is formed during a simulation (Figure C). Mean and error estimated for the temperature dependent population of the dimer phase (main text Fig 4). Since we have 8 independent simulations for each system, the mean and error here are estimated with the Jackknife method by excluding samples from one simulation at a time (Figure D). Free energy of binding for R1 –R5 dimer at the 300K(main text Fig 3) projected to the reaction coordinate D (a) and β-strand content (b). The dashed lines in (a) correspond to D = 10 Å and 15 Å. The dashed line in (b) corresponds to beta (β-strand content) = 0.5 (Figure E). Temperature dependent population of the dimer phase with the D < 10Å(a) and Temperature dependent population of the dimer phase with D < 15Å and β-strand content > 50%(b) (Figure F). The detailed version of the dimer states of R1 (a), R3 (b), R4 (c) and R5 (d) in the main text Fig 6. Nitrogen donor and oxygen acceptor involved in the backbone hydrogen-bonding are illustrated in blue and red, respectively. The intra- and inter- chain hydrogen bonds are shown as yellow dashed line. All the sidechain atoms are not displayed here. The models are drawn with PyMOL (Figure G). (DOCX) [file pone.0146096.s001.docx]

Supporting information

|  |
| --- |
| Figure A. Comparison of secondary structure propensities between calculation and experiment. We show the temperature dependent helical fraction of the (AAQAA)_3_ peptide obtained from previous experiment and simulations using PROFASI force field. |

|  |
| --- |
|  |
| Figure B. Free energy of R1 – R5 monomer at the 300K is projected to the reaction coordinates of the β-strand content (beta) (a) and the α-helix content (alpha) (b). |

|  |
| --- |
| Figure C. Residue contact probability map for R1–R5 (a-e) monomer at 300K. The solid white shows the diagonal of a 19×19 matrix in each plot. The minimum residue distance is 5. Contact maps were constructed based on residue pairs with CA-CA distance below 8Å, containing the percentage of time that this contact is formed during a simulation. |

|  |
| --- |
| Figure D. Mean and error estimated for the temperature dependent population of the dimer phase (main text Fig 4). Since we have 8 independent simulations for each system, the mean and error here are estimated with the Jackknife method by excluding samples from one simulation at a time. |

|  |
| --- |
|  |
| Figure E. Free energy of binding for R1 – R5 dimer at the 300K(main text Fig 3) projected to the reaction coordinate *D* (a) and β-strand content (b). The dashed lines in (a) correspond to D = 10 Å and 15 Å. The dashed line in (b) corresponds to beta (β-strand content) = 0.5. |

|  |
| --- |
|  |
| Figure F. (a) Temperature dependent population of the dimer phase with the D < 10Å. (b) Temperature dependent population of the dimer phase with D < 15Å and β-strand content > 50%. |

|  |
| --- |
| Figure G. The detailed version of the dimer states of R1 (a), R3 (b), R4 (c) and R5 (d) in the main text Fig 6. Nitrogen donor and oxygen acceptor involved in the backbone hydrogen-bonding are illustrated in blue and red, respectively. The intra- and inter- chain hydrogen bonds are shown as yellow dashed line. All the sidechain atoms are not displayed here. The models are drawn with PyMOL. |
